# Investigating Salespeople’s Performance and Opportunistic Behavior: Adaptive and Customer-Oriented Responses

**DOI:** 10.3390/bs12120512

**Published:** 2022-12-15

**Authors:** Chankoo Yeo, Ihsan Ullah Jan

**Affiliations:** 1 Department of International Trade, Kunsan National University, Kunsan 54150, Republic of Korea; 2 Department of Business Administration, Hanbat National University, Daejeon 34158, Republic of Korea

**Keywords:** value congruence, top management support, customer-oriented selling behavior, adaptive selling behavior, sales performance, opportunistic behavior

## Abstract

This study investigates the role of value congruence and top management support on salespeople’s customer-oriented selling behavior and adaptive selling behavior. Moreover, this study has also explored the effects of salespeople’s customer-oriented selling behavior and adaptive selling behavior on sales performance and opportunistic behaviors, respectively. An online survey was administered to collect the data from salespeople in South Korea, and a total of 204 responses were undergone for formal analysis. Partial least squares structural equation modeling (PLS-SEM) was conducted to test the proposed hypotheses. The results showed that salespeople’s value congruence has a significant positive effect on customer-oriented selling behavior and top management support has a significant positive effect on salespeople’s adaptive selling behavior. The salespeople’s customer-oriented selling behavior has a significant positive effect on sales performance and a significant negative effect on opportunistic behavior. Similarly, salespeople’s adaptive selling behavior has significant positive effects on sales performance and opportunistic behaviors. Based on these findings, the implications for theory and practice are discussed in detail.

## 1. Introduction

Salespeople of a company play an important role in communicating the company’s point of view to customers and are instrumental as a medium to provide the product and services of the company in a differentiated way from competitors. In particular, when, in a competitive market, the price per unit of a product becomes more expensive or the characteristics of a product become more complex, the salesperson is not only an agent of the company that sells the product but also a key human factor to deliver a differentiated product image to customers [1,2,3,4]. In fact, salespeople tremendously play a significant role in the fast sensing of market changes, such as the tastes and preferences of the customers during their interactions [5,6]. These roles of salespeople not only increase customer satisfaction with products and companies but also ultimately influence their sales performance [7,8].

In this regard, studies have been conducted on methods for improving salesperson performance through monetary and/or non-monetary compensation. For example, performance-based pay [9], welfare benefits [10], education, training, and effective communication are investigated as monetary or non-monetary determinants of sales performance. However, in the prior literature, researchers have pointed out that monetary and non-monetary compensation is not adequate to increase sales performance [11,12]; therefore, researchers have given attention to the behavioral characteristics of salespeople [13]. Specifically, the behavioral characteristics of salespeople can be influenced by organizational factors as well as trait-related factors of salespeople [14]. For example, the organizational characteristics often encompass sales orientation, values and organizational culture, and customer-oriented selling behavior. Additionally, the individual characteristics comprised of adaptive selling behavior are explored as individual characteristics [14,15].

Looking at related previous studies, many studies considered customer-oriented selling behavior and adaptive selling behavior as similar concepts. However, customer-oriented selling behavior and adaptive selling behavior are different in approach and have effects on salespeople’s outcomes [7,14,15]. For example, customer-oriented selling behavior aims to satisfy customer needs from the customer’s point of view. Therefore, salespeople try to develop a long-term exchange relationship by maintaining a positive relationship with customers, even by neglecting short-term profits. The salespeople only pursue profits that arise from the relationships [16] and avoid taking actions that bring disadvantages to the organization. On the other hand, adaptive selling behavior considers the importance of meeting customer needs, but it is an aggressive sales-oriented behavior to increase short-term sales performance and aims to increase individual salesperson performance [12,17].

Despite these important differences between customer-oriented selling behavior and adaptive selling behavior, related studies are still insufficient to explain the differential effects of these approaches on sales performance and opportunistic behaviors of salespeople. Therefore, the purpose of this study is to examine the differential effects of customer-oriented selling behavior and adaptive selling behavior on sales performance and opportunistic sales behaviors, respectively. Previously, researchers have overlooked examining the influence of salespeople’s customer-oriented selling behavior and adaptive selling behavior and their differential effects on sales performance and opportunistic behaviors in a single framework. In addition, this study investigates the influence of value congruence and top management support on customer-oriented selling behavior and adaptive selling behavior, respectively. Based on the findings of the study, this study intends to present practical implications to appropriately utilize the behavioral characteristics of salespeople in companies such as retail and service industries and to improve their sales performance.

This study is structured as follows. Section 2 of the study provides a comprehensive review of the literature on the key constructs of the study. Section 3 discusses the proposed hypotheses of the study. Section 4 explains the research methodology. Section 5 indicates the findings of the study. Finally, Section 6 offers a detailed discussion of the theoretical and managerial implications as well as the limitations of the study, along with future research directions.

## 2. Literature Review

### 2.1. Customer-Oriented Selling Behavior

In marketing, the success of the company is linked to satisfying customer needs and wants [18]. For this purpose, marketing plays an important role in the identification and satisfaction of customer needs and their purchasing decisions. Specifically, these efforts from the salespeople are called customer orientation [17]. Customer orientation can be seen as an organizational culture that focuses on creating value for customers effectively and efficiently [19] and refers to the values and beliefs shared by organizational members [20]. In other words, it refers to constantly making efforts to satisfy customer needs in terms of identifying customer needs and providing appropriate values [21,22]. These values can be seen as an organizational culture that creates a competitive advantage by identifying the needs and desires of customers and performing activities that satisfy their needs and desires better than those of competitors [22].

Customer-oriented selling behavior is a salesperson’s practice of customer orientation as an action and is influenced by the organization’s culture because it is an action that carries out the organizational culture, values, and marketing philosophy [14,23]. Therefore, it is influenced by the organizational climate [24]. In particular, when a culture that values the value of customer orientation is formed within the organization as a whole [25], sales reps can perform efficient and effective customer-oriented selling behavior to meet customer needs [19].

### 2.2. Adaptive Selling Behavior

Adaptive selling behavior refers to the ability of salespeople to change their sales-related behaviors in the process of forming and interacting with customers [13,26,27] based on perceived information about the sales situation. In other words, it is the ability to take appropriate sales actions for a variety of customers [28]. Therefore, it can be said that the adaptive sales level is high when different sales actions are taken according to different sales situations, and the adaptive sales level is low when the same sales actions are taken in all sales situations [16].

Spiro and Weitz (1990) argued that sales performance differs greatly depending on whether salespeople apply the same method to customers in their relationship with customers or take different approaches to align customer characteristics and circumstances [12]. Saxe and Weitz (1982) argued that salespeople can take adaptive selling behavior regardless of organizational culture or level of the motivational environment [17]. It is judged as a separate selling skill to improve the sales performance of the employee. In other words, adaptive selling behavior can be viewed as an action driven by the individual decision-making of salespeople (working smart). Thus, salespeople who engage in high-level adaptive selling behaviors modify sales presentations based on feedback received from customers.

### 2.3. Value Congruence

Values can be defined as the belief of individuals to perform socially desirable behavior [29,30]. These values are a concept that encompasses what an individual is interested in, wants, and aspire to become. Values are the guiding principles with feelings for individuals’ behavior and are reference points for selecting appropriate means related to the purpose or goals of individuals [31]. Therefore, corporate values in the context of sales demonstrate the specific norms which determine the behavior of salespeople [32]. Value congruence means the degree to which the values held by the salesperson and the company are similar [15]. When the values of the salesperson and the company are shared and similar, the motives and goals of the salesperson and the company are similar, and thus they react in a similar way to events that occur [33]. Therefore, when values are aligned, salespeople can predict the company’s future decision-making through their motivations and goals, reducing uncertainty about the company [29].

In addition, when values are aligned, communication within the company is more effective. The agreement between the salesperson and the corporate values sets a common frame for the explanation, classification, and interpretation of an event by sharing important things with each other, and in this process, communication is actively involved [29]. Active communication facilitates information exchange between salespeople and companies and reduces the possibility of mutual misunderstanding [33]. This reduces uncertainty about the firm [34] and reduces the role ambiguity of salespeople [35].

### 2.4. Top Management Support

Recently, the top management of companies has been emphasizing the importance of salespeople, and they are pursuing the goals of building competitive advantage through salespeople. The CEO’s interest and support affect various areas within the organization. Existing studies have shown that the CEO’s interest and support are related to the knowledge sharing of organizational members and knowledge management [36]. A study by Weitz et al. (1986) reveals that organizational characteristics affect the motivation of employees for their jobs [28]. It can be judged that interest and support can affect the attitudes and sales behavior of salespeople in their jobs. A study by Martin and Bush (2006) explains organizational factors and individual salesperson factors as variables that affect the sales behavior of salespeople [37].

### 2.5. Opportunistic Behavior

Opportunism is the pursuit of one’s own interests through deception [38], which implies a breach of an implicit or explicit promise of appropriate conduct of each party in an exchange relationship. An unexpected situational change [39] or difficulties in understanding a firm’s contractual compliance [40] lead to uncertainty, which in turn increases the level of opportunism of salespeople [41]. Opportunistic behavior refers to the behavior of individuals to manipulate the elements of a company for their advantage [42]. Opportunistic behaviors of salespersons can appear as not exerting their best efforts to achieve corporate goals [43]. In addition, salespeople do not take additional actions related to customer service other than actions that are helpful to themselves, and they take actions such as ignoring sales policies for their own sales performance [43].

These opportunistic behaviors of salespeople cause conflicts in business relationships with customers and may result in contract violations in relationships with organizations [40]. Therefore, it is judged that the level of opportunistic behavior of salespeople will appear differently according to customer-oriented selling behavior that focuses on organizational culture and values and adaptive selling behavior that focuses on individual performance. In the next section, the hypotheses development of the study will be discussed in detail.

### 2.6. Sales Performance

**Figure 1:**
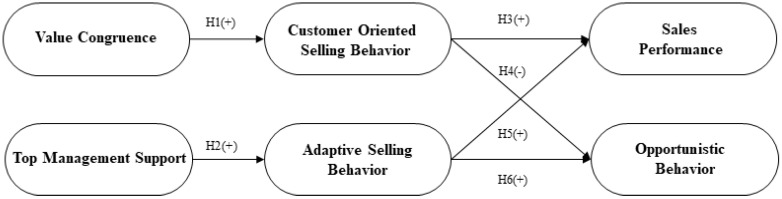
Conceptual model.

Performance is a measure of the effectiveness or efficiency of a company and generally refers to the degree of achievement of quantitative and qualitative goals by employees or sub-departments of the company [44]. The measurement of performance differs depending on the subject of the study because the objectives and structures of each industry are different, so the appropriate performance objectives for the research should be adopted [45]. Oliver and Anderson (1994) divided the sales performance of salespeople into behavioral performance and outcome-based performance [46]. Behavioral performance refers to qualitative performance, such as customer service ability possessed by salespersons and the degree of acquisition of sales-related skills, whereas outcome-based performance is defined as the quantitative performance of salespeople, such as sales or substantial profit. In addition, sales performance includes the contribution and effort of salespeople related to the success of the company [47] and the relationship formation and behavior that can strengthen the relationship that occurs after the customer makes a purchase decision [48]. Figure 1 shows the conceptual model of the study.

## 3. Hypotheses Development and Research Model

### 3.1. Value Congruence and Customer-Oriented Selling Behavior

When salespeople and corporate values align, salespeople become immersed in the company [49]. Researchers have found that salespeople who have the same values as the company (brands) are more likely to take actions that benefit the company even without the formal job description [15,50,51,52]. Moreover, in an environment where values are consistent, salespeople strive to implement essential standard behaviors expected by the organization [53], fulfill their assigned roles in work situations, and voluntarily perform additional extra-role behaviors [54].

The incongruence of the values of the organization to the salespeople increases the role ambiguity of salespeople [55], which may act as a cause of failure to perform appropriate sales behavior [56]. In other words, if the salesperson and the company’s values match, the salesperson’s immersion in the company increases, demonstrating excellent work performance and striving to implement the standard behavior set by the company. Therefore, the current study proposes the following:

**Hypothesis 1.**  *The congruence of the salesperson’s value and the company’s value will have a positive effect on the salesperson’s customer-oriented selling behavior*.

### 3.2. Top Management Support and Adaptive Selling Behavior

Support from top management influences the sales behavior of salespeople, which is generally expressed through delegation of authority [13]. Delegating authority to salespeople by the top management includes expressing trust, creating an environment that enhances self-efficacy and control, setting realistic and high expectations, eliminating factors that cause feelings of helplessness, and allowing freedom to do things flexibly [57,58].

Salespeople who have been granted this authority by the top management can make decisions with sufficient motivation and confidence to carry out sales [59] and have the flexibility to handle their work and better coordinate their sales behavior [60]. Therefore, salespeople who have secured autonomy and motivation for decision-making due to the CEO’s delegation of authority regulate their own actions and actively participate in their roles based on the decision-making authority and autonomy given to them to perform sales more adaptively [61]. Based on this perspective, it can be predicted that top management support affects the adaptive selling behavior and salespeople, and the following hypothesis is proposed;

**Hypothesis 2.**  *The top management’s support of salespeople will have a positive effect on their adaptive selling behavior*.

### 3.3. Customer-Oriented Selling Behavior and Sales Performance

Prior studies show that effective salesperson behavior leads to increased salesperson and corporate performance [46,62,63]. A salesperson’s customer-oriented selling behavior is the salesperson’s efforts to identify and satisfy the needs of target customers to achieve long-term goals [64]. Salespeople with high sales performance identify customer situations and needs, establish a sales plan, execute efficient sales to customers, and respond quickly to customer needs during interaction [65]. In other words, salespeople who accurately understand customer needs and execute sales strategies have higher sales performance. Franke and Park (2006) [66] and Martin and Bush (2006) also argued that the customer-oriented selling behavior of salespeople increases sales performance [37]. Based on these previous studies, the current study proposes the following:

**Hypothesis 3.**  *Customer-oriented selling behavior of salespeople will have a positive effect on sales performance*.

### 3.4. Customer-Oriented Selling Behavior and Opportunistic Behavior

Opportunism can be defined as the tendency of humans to secretly pursue their own interests against the will of others [67]. This is a human tendency to strategically distort transaction-related information or falsely express one’s intentions to pursue profits from an advantageous position over the other party [43].

In the marketing context, opportunism has been mainly investigated in the context of the power exercise of business-to-business relationships, particularly the distribution channels. Generally, a partner’s opportunism deteriorates the mutual trust and cooperative spirit between partners, increasing risk perception and ultimately worsening the relationship between channel members. Consequently, salespeople who embodied in a customer-oriented organizational culture will feel a sense of responsibility and will act in line with the organizational culture that prioritizes long-term and trusting relationships with customers (working hard). As such, the customer-oriented selling behavior of salespeople can be predicted to have a negative effect on opportunistic behavior because of the inherent organizational customer orientation, and thus the following hypothesis is proposed:

**Hypothesis 4.**  *A salesperson’s customer-oriented selling behavior will have a negative effect on opportunistic behavior*.

### 3.5. Adaptive Selling Behavior and Sales Performance

In the prior literature, studies have shown contradictory findings on the relationship between selling behavior and sales performance. Johnson et al. (2009) found a positive relationship between adaptive selling behavior and sales performance [68]. Guenzi et al. (2009) argued that there is a negative relationship between the two variables [69], and Boles et al. (2001) argued that there was no significant relationship between adaptive selling behavior and sales performance [70]. Yi (2017) identified the cause of the differentially derived influence relationship between adaptive selling behavior and sales performance as a problem in the industrial environment in which the study was conducted [71]. In this respect, the effect between adaptive selling behavior and performance can be expected to happen only under certain conditions. For example, Saxe and Weitz (1982) judged that adaptive selling behavior was effective when customers had low repeated purchase frequency; salespeople had low professionalism, and given tasks were simple [17]. Dweck and Leggett (1988) found sales-oriented selling since employees are sensitive to compensation, it is argued that if an appropriate evaluation system and compensation system are in place in a given situation, they will work hard to obtain monetary compensation and recognition within the organization, which will result in tangible results [72].

On the other hand, studies dealing with the relationship between adaptive selling behaviors and sales performance as a specific sales technique generally suggest a consistent direction that adaptive selling behavior has a positive effect on sales performance [12,73,74]. Park and Holloway (2003) confirmed that adaptive selling behavior has an important effect on sales performance or job satisfaction in the context of automobile salespeople [75]. Porter et al. (2003) also confirmed the relationship between adaptive selling behavior and sales performance [76]. Therefore, it can be predicted that sales performance will be affected by adaptive selling behavior, and the following hypothesis is proposed.

**Hypothesis 5.**  *A salesperson’s adaptive selling behavior will have a positive effect on sales performance*.

### 3.6. Adaptive Selling Behavior and Opportunistic Behavior

In adaptive selling behavior, salespeople modify sales strategies according to customer needs and sales conditions. Because of that, in some cases, adaptive selling behavior lacks personal consideration for customers and damages the quality of relationships with customers [77].

As such, adaptive selling behavior is closely related to opportunistic behavior because they prioritize their short-term performance rewards over the long-term interests of the organization [12]. In other words, salespeople are more likely to take opportunistic actions to increase their own profits if they meet the purpose of increasing effectiveness (working smart) in the pursuit of profit. Therefore, it can be predicted that the opportunistic behavior of the salesperson will be affected by the adaptive selling behavior, and the following hypothesis is proposed.

**Hypothesis 6.**  *A salesperson’s adaptive selling behavior will have a positive effect on opportunistic behavior*.

## 4. Methodology

### 4.1. Sample and Data Collection

A quantitative survey method is used to test the hypothesized relationships of the study indicated in Figure 1. The data for the study was collected from the salespeople of different industries in South Korea using an online survey. The survey was initially prepared in English and then translated into the Korean language by using the back-translation method. The back-translation approach is a highly recommended approach to translating the scale from one language to another language [78]. Specifically, the scale from the English language is translated into Korean language and then subsequently translated back into the English language to evaluate the accuracy of the translation. In order to increase the representativeness of salespeople for this study, screening questions were asked before asking the main questions of the study. There were five screening questions. The first question was, “are you currently working as a salesperson?”; if the answer was “Yes”, then the respondent was allowed to move to the next questions, which were “What is your company name?”, “Name of the department?”, “How long have you been in your current department?” and finally, “What is your main product/service name?”.

A total of 215 samples were collected. A total of 9 cases were eliminated based on the missing data, which resulted in a sample of 204 for final analysis. Among the 204 respondents, 120 (58.8%) were females and 84 (41.2%) were male. Based on the age groups, the largest number of the sample came from the age group in their 30s, who were 84 (41.1%) of the respondents of the sample. Similarly, 51 (25%) of respondents came from the age group of the 40s. Based on the industry, most of the respondents were from the fashion 43 (21.1%) and food and drink industry 40 (19.6%). Table 1 shows detailed information about the demographic characteristics of the respondents.

### 4.2. Measurements

In order to measure the constructs of the current study, pre-validated scales were taken and adapted from the prior literature. The constructs were measured on a five-point Likert scale where 1 stands for strongly disagree and 5 for strongly agree. Specifically, five items were used to measure value congruence taken and adapted from the study [79]. Top management support was measured by four items taken and adapted from the study of [80]. Adaptive selling behavior was measured by five items taken from Spiro and Weitz (1990) [12]. Customer-oriented selling behavior was measured by four items taken and adapted from Saxe and Weitz (1982) [17]. The sales performance of the salespeople was measured by four items taken from [81]. Finally, the opportunistic behavior was measured by four items taken and adapted from [82]. All the measurement items of the constructs are given in Table 2 in detail.

### 4.3. Common Method Bias

Since the data for the independent and dependent variables were collected at a single point in time, it increases the likelihood of common method bias. The issue of common method bias can affect the results. Therefore, before conducting the formal data analysis, we tested the common method bias by using Harmon’s single-factor test in SPSS. We loaded the items of constructs in a single factor with an un-rotated factor solution. The results showed that a single factor accounted for 34.17% variance, which is less than 50% of the threshold value, confirming the absence of common method bias.

The inner variance inflation factor (VIF) values are calculated to test the multi-collinearity. We found that the values of VIF are less than the cutoff of 3.3, which indicates the absence of multi-collinearity issues [83]. Table 2 summarizes the inner VIF values of the constructs.

## 5. Results

### 5.1. Reliability and Validity

To empirically test the proposed hypotheses of the study, we used variance-based structural equation modeling (SEM) via Smart-PLS 3.0. PLS-SEM is a powerful multivariate analysis technique that is used to estimate more complex models [84]. In the prior literature, researchers have used two-step analyses for structural equation modeling [85]. Firstly, the confirmatory factor analysis is conducted to evaluate the reliability and validity of the measurement model, and subsequently, in the second step, the structural model is tested.

The results of the measurement model assessments are provided in Table 3. The standard factor loadings of the measurement items were above the threshold value of 0.70 [86]. The values of Cronbach’s alpha and composite reliability were above 0.70, which showed the reliability of the scale. The values of average variance extracted (AVE) of the constructs are above 0.50, reflecting the convergent validity of the constructs [86,87]. The discriminant validity of the constructs is also tested by comparing the square root of AVE for each construct to the coefficients of inter-construct correlations. The square roots of AVE are higher than the constructs’ inter-construct correlations, which validated the discriminant validity of the scale [88]. Table 4 indicates the coefficients of the correlation matrix and square roots of AVE in a diagonal.

We calculated the Heterotrait-monotrait (HTMT) ratio to further validate the discriminant validity of the measurement model. The results of the HTMT analysis are shown in Table 5. All the values are below the benchmark of 0.85 [89], establishing the discriminant validity.

### 5.2. Hypotheses Testing

To test the proposed hypotheses of this study, we applied PLS-SEM of 5000 bootstrapping of 204 cases. The results of the hypotheses testing are given in Table 6. H1 of the study is supported as the positive relationship between value congruence, and customer-oriented selling behavior was significant (β = 0.376, *p* < 0.01). Similarly, the results showed that top management support has a significant positive effect on adaptive selling behavior (β = 0.464, *p* < 0.01); thus, the H2 of the study is supported. H3 of the study proposed a positive relationship between customer-oriented selling behavior and sales performance which is also supported (β = 0.215, *p* < 0.01). H4 of the proposed positive relationship between adaptive selling behavior and sales performance is also supported (β = 0.554, *p* < 0.01). The proposed negative relationship between customer-oriented selling behavior and opportunistic behavior was supported (β = −0.352, *p* < 0.01); thus, H5 of the study was supported. Finally, H6 of the study supported as the positive relationship between adaptive selling behavior, and opportunist behavior was significant (β = 0.311, *p* < 0.01). The results showed that the coefficient of determination (R^2^) for customer-oriented selling behavior is 0.131, adaptive selling behavior is 0.212, sales performance is 0.476, and opportunistic behavior is 0.092. We found that value congruence explains 13.1% of customer-oriented selling behavior, and top management support explains 21.2% of adaptive selling behavior. Similarly, customer-oriented selling behavior and adaptive selling behavior explain 47.6% of sales performance and 9.2% of opportunistic behavior.

## 6. Discussion

In this study, we discussed the differentiated characteristics of customer-oriented sales behavior and adaptive sales behavior on sales performance and opportunistic behavior. Customer-oriented sales behavior views the relationship with customers as a long-term-oriented relationship and focuses on continuous performance rather than short-term performance. Adaptive sales behavior focuses on short-term relationships to achieve high performance based on flexibility in sales behavior. Focusing on short-term relationships and trying to improve performance can also lead to opportunistic behavior.

Previously, some studies have argued that adaptive sales behavior has a positive effect on sales performance [12,73,74,75]. Hence, the findings of the studies are consistent with the previous studies, which showed that adaptive selling behavior contributes to sales performance positively. On the contrary, the results of this study revealed that adaptive selling behavior leads to opportunistic behavior. These findings are new to the literature on opportunism in the context of sales. As previously discussed, the researchers have mainly investigated opportunism in the context of distribution channels.

Similarly, customer-oriented selling behavior has a positive effect on sales performance which is consistent with the previous studies [66]. However, the results of the study showed that customer-oriented selling behavior is negatively related to opportunistic behavior which are new findings of the study.

### 6.1. Theoretical Implications

The theoretical implications of this study can be summarized as follows. First, in this study, the behavioral characteristics, such as customer-oriented selling behavior and adaptive selling behavior of salespeople, are examined in relation to the sales performance and opportunistic behavior of salespeople. Unlike the previous studies, the antecedent factors affecting the behavioral characteristics of salespeople were comprehensively examined without distinction of the behavioral characteristics specifically the customer-oriented selling behavior and adaptive selling behavior [13,14]. Moreover, although there are many studies on the behavioral characteristics of salespeople that affect outcome factors, studies on the common or differential effects of variables are insufficient. Therefore, this study suggests the theoretical implications of identifying the common or differential effects of the salesperson’s behavioral characteristics on the salesperson’s behavioral outcomes and sales performance that can appear at the point of contact providing services.

Second, looking at the existing studies, most of the studies show that adaptive selling behavior has a positive effect on organizational performance [14]. In this study, adaptive selling behavior was also found to have a positive effect on the sales performance of a company as a representative variable of sales orientation, but it was also found to have a positive effect on the opportunistic behavior of salespeople. This has great significance in that it revealed that there were also negative effects, unlike previous studies that identified positive effects on adaptive selling behavior. The opportunistic behavior of salespeople shown in this study has great implications for many companies that present sales performance as an evaluation index. In the short term, improving the sales performance of the organization brings great benefits to the organization, but the salesperson’s adaptive selling behavior to improve the short-term sales performance can cause great damage to the organization in the long term. Therefore, it suggests that the organization needs to analyze the effect of sales-oriented factors on the organization from more diverse aspects.

### 6.2. Managerial Implications

This study provides the following practical implications. First, customer-oriented selling behavior is a characteristic of salespeople that reflects the organizational culture that most effectively and efficiently identifies the needs and desires of customers to create value for customers. Before doing so, it is necessary to form an organizational culture that puts customer value first by providing a variety of education. Recently, it can be seen that many companies are implementing CS (customer satisfaction) education for their salespeople. However, after the outbreak of the COVID-19 virus, education related to customer orientation is also changing because of social distancing. This is because, as the number of industries that sell products non-face-to-face and start online consultations, customer-oriented activities that create customer value are also being conducted online. Therefore, it is suggested that companies need to better understand the needs and desires of customers at the time of online sales and introduce a training course that can improve customer satisfaction.

Second, value congruence can be used as a way to manage salespeople, which is one of the important channels of a company. Salespeople play a key role in building relationships with customers as well as sales. Considering the roles of these salespeople, it is very important to manage salespeople according to their goals for the success of a company. Based on the vision pursued by the company, by fully sharing the company’s values and goals with the salespeople, the values of the company and the salespeople can be aligned, thereby strengthening the behavior of the salespeople desired by the company. To match the values of the salesperson and the company, value education should be established in the salesperson training system. It is important to make salespeople feel the necessity of values and to form a consensus on the values through continuous education about corporate values. For this, education related to values should be included in salesperson training and workshops. In other words, it is necessary to educate all salespeople to increase their understanding of corporate values and create an environment for salespeople to share values by strengthening their awareness of corporate values, especially for managers.

### 6.3. Limitations and Future Research Directions

Although this study has found significant results for all hypotheses, however, there are following limitations. First, this study is expected to have limitations in generalizing the results because the survey was limited to salespeople engaged in the service industry. Therefore, in future research, it is necessary to increase the generalizability of the results by collecting data from various other industries.

Second, this study identified the difference between the two sales behaviors, customer-oriented selling behavior and adaptive selling behavior, but indirectly confirmed the difference based on the opportunistic behavior level. However, to confirm a more fundamental difference between the two sales behaviors, a more direct method should be used, such as analyzing sales data or comparing the short-term/long-term performance between the two sales behaviors by introducing a time series analysis. Therefore, it will be possible to increase the theoretical validity of the results of this study by attempting to explore this avenue in future studies.

Third, this study only confirmed the difference between the two sales behaviors, and the possibility of interaction between the two sales behaviors was not considered in this study. Although there are differences between the two selling behaviors, they are not mutually exclusive strategies because they also have commonalities [66]. Therefore, they can influence each other, and it will be possible to study the situation in which two sales behaviors are used at the same time and the influence they have at that time. For example, there may be differences in the effect according to the order, including the possibility of starting with adaptive sales and switching to customer-oriented selling when the number of years of service with a specific customer is long, or vice versa. Furthermore, in a specific customer group, even in a relationship with the same customer, both strategies may be used simultaneously, such as customer-oriented selling behavior and adaptive selling behavior, depending on the situation. Therefore, it is another area that needs a follow-up study to explore.

Finally, previous studies on the two sales behaviors revealed that organizational traits, including individual psychological traits of salespeople, affect not only sales propensity but also sales performance [74,90]. Therefore, among these variables, it is expected that there will be variables that can change the results of this study. For example, more relationship-oriented people may prefer customer-oriented selling behavior over adaptive sales behavior because they value relationships and are expected to have lower opportunistic tendencies. Therefore, future research can also examine this perspective too.

## Figures and Tables

**Table 1:** Demographic characteristics.

Demographic Characteristics	Frequency	Percentage
**Gender**		
Male	84	41.2%
Female	120	58.8%
**Age**		
20–29	49	24.1%
30–39	84	41.1%
40–49	51	25.0%
50–59	20	9.8%
**Job Position**		
Employee	108	52.9%
Chief	34	16.7%
Assistant Manager	29	14.2%
Manager	16	7.8%
Assistant Director and Director	17	8.4%
Industries		
Fashion	43	21.1%
Food and drink	40	19.6%
Home appliance	25	12.3%
Restaurants	18	8.8%
Beauty products	16	7.8%
Insurance and bank	13	6.4%
Cultural performance service	7	3.4%
Hospital	7	3.4%
Hotel	6	2.9%
Others	29	14.3
Total	204	100%

**Table 2:** Inner VIF values.

Constructs	ASB	COSB	OPP	PER
1. ASB			1.409	1.409
2. COSB			1.409	1.409
3. TMC	1.000			
4. VC		1.000		

ASB: adaptive selling behavior, COSB: customer-oriented selling behavior, OPP: opportunistic behavior, PER: sales performance, TMS: top management support, VC: value congruence.

**Table 3:** Measurement items and the results of confirmatory factor analysis.

Constructs	Measurement Items	Loadings	α	CR	AVE
Value congruence	VC1. My values match with the values of the company.	0.870	0.908	0.931	0.731
	VC2. The values that the company considers important reflect my values.	0.884			
	VC3. I think the values of this company are desirable.	0.856			
	VC4. I would not have joined this company if my values were different from the values of this company.	0.827			
	VC5. The reason why I chose my current company is because of its values.	0.839			
Top management support	TMS1. Company management takes the interest of salespeople seriously.	0.829	0.893	0.926	0.758
	TMS2. Company management puts a lot of effort into improving the well-being of salespeople.	0.894			
	TMS3. Company management strives to develop individual salespersons and professionalism.	0.877			
	TMS4. The company’s management gives importance to the job satisfaction of salespeople.	0.880			
Customer-oriented selling behavior	COSB1. Our company expresses commitment to our customers.	0.804	0.838	0.891	0.672
	COSB2. Our company creates value for our customers.	0.761			
	COSB3. Our company understands the needs of our customers.	0.862			
	COSB4. Our company sets customer satisfaction objectives.	0.849			
Adaptive selling behavior	ADS1. I sell products or services in a very flexible way.	0.706	0.855	0.896	0.634
	ADS2. I use a variety of sales methods easily.	0.822			
	ADS3. I do not use the same approach for the most of customers.	0.807			
	ADS4. I like to try various sales approaches to the customer.	0.852			
	ADS5. I try to change my approach according to each customer.	0.787			
Sales performance	SP1. I am increasing the market share of the company in a certain area.	0.842	0.884	0.920	0.743
	SP2. I am increasing the sales volume of a product or service.	0.887			
	SP3. I am selling new products or services quickly.	0.860			
	SP4. I am finding new customers and forming relationships.	0.859			
Opportunistic behavior	OPB1. I promise my boss that I will do it without doing anything.	0.875	0.912	0.933	0.737
	OPB2. I intentionally exaggerate for sales opportunities in certain situations.	0.839			
	OPB3. Sometimes I distort the facts to some extent to get what I need.	0.872			
	OPB4. If I have an opportunity to make an operating profit, I may not keep my promises or contracts with the company.	0.886			
	OPB5. I sometimes inconsistently carry out agreements with the company.	0.820			

**Table 4:** Correlation matrix and squared roots of AVE in diagonal.

Constructs	1	2	3	4	5	6
1. ASB	**0.796**					
2. COSB	0.539	**0.820**				
3. OPP	0.116	−0.186	**0.859**			
4. PER	0.666	0.510	0.115	**0.862**		
5. TMS	0.461	0.366	0.017	0.364	**0.870**	
6. VC	0.479	0.368	0.131	0.400	0.697	**0.855**

ASB: adaptive selling behavior, COSB: customer-oriented selling behavior, OPP: opportunistic behavior, PER: sales performance, TMS: top management support, VC: value congruence.

**Table 5:** HTMT.

Constructs	1	2	3	4	5	6
1. ASB						
2. COSB	0.630					
3. OPP	0.138	0.222				
4. PER	0.762	0.579	0.134			
5. TMS	0.522	0.424	0.076	0.409		
6. VC	0.538	0.404	0.154	0.441	0.774	

ASB: adaptive selling behavior, COSB: customer-oriented selling behavior, OPP: opportunistic behavior, PER: sales performance, TMS: top management support, VC: value congruence.

**Table 6:** Results of the structural model.

H	Hypotheses	β	T-Statistics	*p*-Value	CL (5–95%)	Results
H1	Value congruence → customer-oriented selling behavior	0.376	6.143	0.000	0.274—0.471	Supported
H2	Top management support → adaptive selling behavior	0.464	8.375	0.000	0.373—0.554	Supported
H3	Customer-oriented selling behavior → sales performance	0.215	2.762	0.003	0.086—0.340	Supported
H4	Adaptive selling behavior → sales performance	0.554	9.518	0.000	0.456—0.648	Supported
H5	Customer-oriented selling behavior → opportunistic behavior	−0.352	4.407	0.000	−0.479—0.220	Supported
H6	Adaptive selling behavior → opportunistic behavior	0.311	3.799	0.000	0.178—0.443	Supported

## Data Availability

Not applicable.
